# Posterior component separation with transversus abdominis muscle release versus mesh-only repair in the treatment of complex ventral-wall hernia: a randomized controlled trial

**DOI:** 10.1186/s12893-022-01794-7

**Published:** 2022-09-20

**Authors:** Mohamed Rabie, Mahmoud Abdelnaby, Mosaad Morshed, Mostafa Shalaby

**Affiliations:** Colorectal Surgery Unit, Department of General Surgery, Mansoura University Hospitals, Mansoura University, 60 ElGomhouria Street, Mansoura, 35516 Dakahliya Egypt

**Keywords:** Hernia, Complex, Ventral-wall, Mesh, Sublay, Posterior component separation, Transverses abdominus release

## Abstract

**Background:**

Complex ventral hernias (VHs) represent a real challenge to both general and plastic surgeons. This study aims to compare Sublay Mesh-Only Repair to Posterior Component Separation “PCS” with Transversus Abdominis Release “TAR” in the treatment of complex ventral-wall hernias (VHs).

**Methods:**

This a randomized, controlled, intervention, including two parallel groups: A; Sublay Mesh-Only Repair and Group B; “TAR”. Consecutive patients of both genders aged between 18 and 65 years old with complex VHs presented at Mansoura University Hospitals including large-sized abdominal-wall hernia ≥ 10 cm in width, loss of domain ≥ 20%, multiple hernial defects, or recurrent hernias. Immuno-compromised patients, patients with liver impairment, or severe heart failure were considered an exclusion criterion. The primary outcome is the recurrence rate after 12-months following the procedure.

**Results:**

Fifty-six patients were recruited in this study. There was no significant difference between both groups regarding recurrence. However, there was significant differences between both groups regarding seroma favoring mesh-only repair.

**Conclusions:**

Although TAR may be associated with longer operative times and more blood losses, these were not found to be statistically significant. Postoperative complication, except for seroma, and recurrence rates were comparable in both groups.

*Trail registration* The study was registered on clicaltrials.gov “NCT04516031”.

## Introduction

Complex ventral hernias (VHs) represent a real challenge to both general and plastic surgeons. VHs are considered complex when certain criteria are found including but not limited to; hernia defect > 10 cm, loss of domain > 20%, hernia present over a bony prominence, burst abdomen, and multiple hernial defects. The complexity of hernia increases the perioperative measures, risks, and complications [1].

Although mesh repair improves the outcome by decreasing the recurrence, it affects the flexibility of the abdominal wall especially in case where large sheets of mesh are used. Other complications include surgical site infection (SSI), the formation of adhesions, and the occurrence of enterocutaneous fistulae [2].

Many refinements have been added to decrease the recurrence rate and mesh-related complications. These techniques differ in the anatomical placement of the prosthesis in relation to the muscle: onlay (superficial to the muscles), inlay (interposition), sublay (retro-rectus), underlay (pre-peritoneal), or intra-abdominal (intra-peritoneal) [3].

The Rives-Stoppa technique was introduced, in which the prosthesis is placed as an extraperitoneal deep to the transversalis fascia and superficial to the peritoneum with wide overlapping (> 10 cm) with the facial defect. This allows a tension-free closure of the defect and a wider surface area for tissue ingrowth through the implanted mesh [4–7]. However, the plane is limited by the lateral border of the posterior rectus sheath which is the case in larger abdominal-wall defects [8].

The posterior component separation (PCS) technique is considered a modification of the Rives-Stoppa technique. It allows the development of an intramuscular plane between the posterior rectus sheath and rectus abdominis muscle to permit placement of a mesh in a sublay fashion. This space can be increased by transversus abdominis muscle release (TAR) allowing significant medial advancement of the posterior rectus fascia while preserving the neuromuscular supply and provides a wider space for sublay-mesh placement [9]. Novitsky et al. [10] in 2012 published the technical details of the PCS with TAR that provides a durable hernia repair with a lower rate of recurrence, limited significant wound morbidity, rare mesh-related complications, and no instances of complete explantation of the mesh.

PCS with TAR has rapidly gained popularity in local myofascial advancement. Several studies have reported its value in complex VHs, to the best of our knowledge this will be the first randomized controlled trial to compare it to mesh-only repair. We hypothesized that TAR would be safe and effective. This study is a randomized controlled intervention with two groups; mesh-only repair and PCS with TAR in the treatment of complex VHs. The primary outcome is the 1-year’s recurrence rate.

## Patients and methods

### Study design

The design adopted for this study was a prospective randomized, controlled, intervention, including two parallel patient-groups, with the primary outcome was 1-year recurrence following the initial treatment. The study was registered on clicaltrials.gov “NCT04516031”.

### Ethics approval and guidelines, participants, inclusions, and exclusions criteria

As a routine at our institute, all experimental protocols were approved by Institutional Review Board in Mansoura University and all methods were carried out in concordance with the Helsinki Declaration Principals. An informed consent was obtained from every participant before enrollment in the study. On request, patients were able to be excluded, at any time, from the study if they did not want to continue. The data was then collected and analyzed prospectively.

The patient’s recruitment process was started and continued from September 2018 to October 2019. Consecutive patients of both genders aged between 18 and 65 years old presented at the general surgery outpatient departments at Mansoura University Hospital with complex VHs adopted from the Slater et al. [1] definition were recruited for the study. Four criteria were employed for selection; (1) large-sized abdominal-wall hernia ≥ 10 cm in width, (2) loss of domain ≥ 20%, (3) multiple hernial defects, or (4) recurrent hernias, at least one criterion should be fulfilled to be considered complex. Immuno-compromised patients, patients with liver impairment, or severe heart failure were considered an exclusion criterion.

### Pre-enrollment

After careful history taking and thorough clinical examination, the diagnosis of VH was achieved. Pelvi-abdominal ultrasound (US) was routinely ordered to comment on defect size. All patients were seen routinely in the pre-assessment clinic by an anaesthetist. The day before surgery the patient received adequate thromboembolic prophylaxis with low molecular weight heparin (LMWH) if indicated. At the time of anaesthesia induction, antibiotic prophylaxis with 1.5 gm Ampicillin/Sulbactam was administered.

### Interventions

Eligible patients were randomized equally between Group A “Mesh-Only Repair” and Group B “PCS with TAR”.

#### Surgical technique

In both techniques, the skin and the subcutaneous fat of the midline were incised then any visceral adhesion to the anterior abdominal wall and pelvis were fully lysed.

##### Mesh only repair

The mesh-only “sublay” repair was done as described by Wantz [11] in 1991. Incision of the posterior rectus fascia was performed close to the linea alba and a pocket was then created posterior to the rectus abdominis muscle and anterior to the posterior rectus fascia and the peritoneum. The peritoneum and the posterior rectus fascia were closed with a running absorbable suture (Polysorb™ round Suture, Medtronic® Minneapolis, MN, USA) to form a barrier between the implanted mesh and the abdominal contents. If the tissue is insufficient to close this layer, a piece of absorbable mesh (Vicryl^®^ Woven Mesh, Ethicon® Somerville, NJ, USA) or an incorporated piece of omentum was used to fill the gap.

The mesh was placed underneath the rectus muscle through its full width overlapping above and below the margins of the fascial defect by 4–6 cm. The mesh was then fixed by non-absorbable sutures (Surgipro™ round Suture, Medtronic^®^ Minneapolis, MN, USA) through the abdominal wall. The mesh was laid wrinkle-free as much as possible. Closed suction drain were placed anterior to the mesh. The anterior rectus fascia, linea alba, and scar tissue were then approximated in the midline to cover the mesh and to isolate it from the subcutaneous tissue. The subcutaneous tissue was sutured and the skin was then closed by stapler or sutures.

##### PCS with TAR

**Fig. 1:**
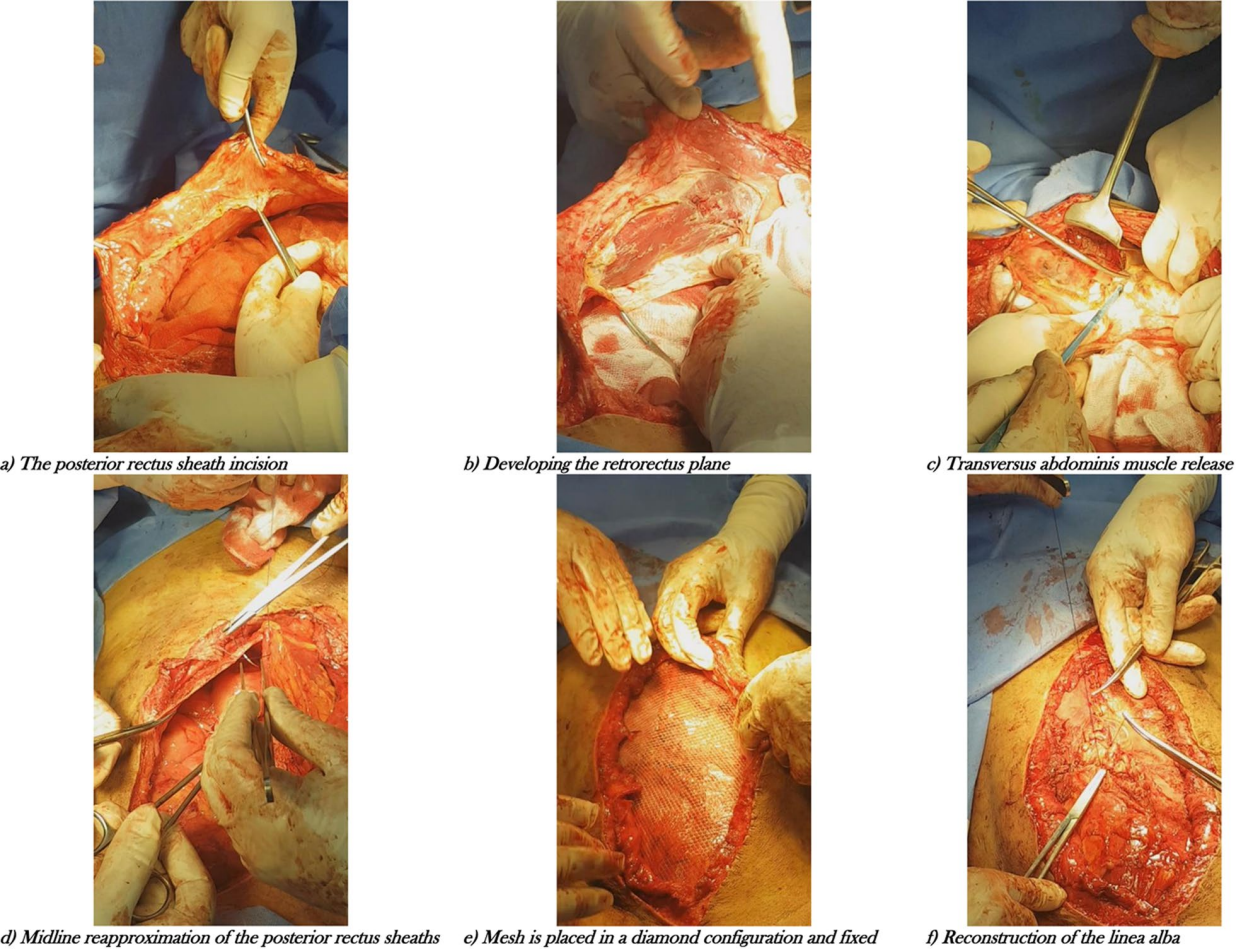
**a** The posterior rectus sheath incision. **b** Developing the retrorectus plane. **c** Transversus Abdominis Muscle release. **d** Midline reapproximation of the posterior rectus sheaths. **e** Mesh is placed in a diamond configuration and fixed. **f** Reconstruction of the linea alba

The PCS with TAR was performed as prescribed by Novitsky et al. [10] in 2012. An incision was then made in the posterior rectus sheath within 0.5 cm of its medial border through the entire length of the rectus muscle and it was then dissected from the muscle to develop a retromuscular plane (Fig. 1a, b). The posterior rectus sheath was incised, approximately 0.5–1 cm medial to the neurovascular bundle to rectus abdominis muscle and linea semilunaris and the transversus abdominis muscle was transected along its whole length. Once divided, the muscle was anteriorly retracted to develop an avascular retromuscular plane (Fig. 1c). On the contralateral side, the same was done.

The posterior rectus sheath was reapproximated in the midline with all holes in the posterior layer closed (Fig. 1d). The mesh (Surgipro™ Polypropylene mesh Medtronic^®^ Minneapolis, MN, USA) was then placed in a diamond configuration (Fig. 1e) and fixed with a trans-fascial suture (Surgipro™ round Suture, Medtronic^®^ Minneapolis, MN, USA) just above the pubic ramus and around the xiphoid process. On each side, full-thickness trans-fascial sutures were placed in three cardinal points in a physiological tension. The linea alba was reconstructed by suturing the anterior rectus sheaths to each other in the midline (Fig. 1f). Closed suction drain was placed anterior to the mesh. The subcutaneous tissue was closed in layers then the skin was stapled or sutured.

#### Postoperative care

Patients were encouraged for early ambulation and early feeding, once the intestinal motility recovered, clear oral fluid was started. Ampicillin/Sulbactam 1.5 gm was continued for more three doses, however, LMWH was continued only for those who had thromboembolic risk. Bedside clinical parameters were recorded every 6 h. Wounds were inspected daily for seroma, hematoma, and infection. The drain was monitored for amount and colour, later on, it was removed after 7 days or if the daily output decreased to 50 ml/day. Patients were discharged after tolerating an adequate oral diet with the pain controlled by oral analgesics, provided that they were free from any complications. Patients were instructed to avoid heavy lifting for a period of 4 weeks.

### Patient’s follow-up

All patients were followed up in the outpatient department for a period of 12 months. The schedule of follow-up was at the POD 7th, POD 15th, then at the 1, 3, 6, and 12 months after the procedure. In case of any adverse events related to the procedure that occurred during the trial, patients were advised to visit the outpatient department without waiting for the next follow-up appointment.

At every visit, thorough clinical examination was performed and if recurrence was suspected, a complementary pelvi-abdominal (US) or Computed Tomography (CT) were indicated. Due to the impact of the COVID-19 pandemic with the suspension of elective clinical visits, follow-up was conducted and continued via a telephone-call, and patients with suspected recurrence had an appointment arranged for assessment.

### Outcomes and definitions

The primary outcome was the recurrence rate after 1 year following the procedure. Secondary outcomes included operative time; estimated blood loss; mesh size; the number of drains and time of removal; pain score; length of hospital stay; postoperative morbidity including wound and mesh-related complications such as seroma, hematoma, infection, and wound grade.

The pain was measured at the night of operation and on the 1st POD using the Visual Analog Scale (VAS) ranged from no pain “0” to worst pain “10”. We adopted these definitions; for seroma (accumulation of fluid in the site of the operation for which surgical intervention either aspiration or drainage was required), for hematoma (accumulation of blood in the field of operation for which surgical intervention either aspiration or drainage was required).

The wound was graded by the Center for Disease Control scoring; I: normal wound, II: erythema and swelling, III: purulent effluent and IV: open wound. Postoperative morbidity was classified according to the Clavien–Dindo Classification [12].

### Sample size calculation

The sample size was 28 participants for each group of intervention. Given an expected medium effect size of 0.5 and P < 0.05, the acceptable power of 0.80 is obtained. The sample size was calculated using online software (http://clincalc.com/stats/samplesize.aspx) with the recurrence rate for “Mesh-Only Repair” was 32% according to Burger et al. [13] and the recurrence rate for “PCS with TAR” was 3.7% according to Novitsky et al. [14].

### Randomization, allocation, and blindness of the trial

All participants who gave consent and who fulfilled all inclusion criteria were randomly assigned to either “Mesh-Only Repair” or “PCS with TAR”. The randomization schedule was generated by an online software (Research Randomizer Version 4.0 at https://www.randomizer.org). The randomization plan was formed of 2 sets; each set contained 28 unique numbers arranged from the smallest to the largest with the whole 56 numbers ranging from 1 up to 56. Each set was labelled with the intervention name, in case more than one patient was operated on the same day, a numbered, opaque, sealed envelopes which include the type of intervention were employed. The randomization plan was performed one in advance before starting the requirement process by one of the investigators, however, opening sealed envelopes and assign patients to their allocated intervention was taken by a resident who did not take part in the study. This study was conducted in an open-label fashion in which the participants, surgeons, investigators, and assessors were aware about the intervention taken.

### Variables studied and statistical analysis

Patient-related parameters included age, sex, body mass index (BMI) status, American Society of Anesthesiology (ASA) class, any associated comorbidities, and any prior abdominal surgery. Hernia-related parameters included the duration of symptoms in months; nature of hernia whether primary or recurrent; type of the hernia whether epigastric, paraumbilical, umbilical, or incisional; and the size of the defect on abdominal imaging.

Operative-related parameters included type of the intervention and size of the mesh used; method of mesh fixation; operative time in minutes; estimated operative blood loss in ml; the number of placed drains; and the method of skin closure. Outcomes-related parameters included the recurrence rate after 1 year follow-up; pain score; length of hospital stay; time of drains’ removal; and postoperative morbidity.

The data were analyzed using SPSS (Statistical Package for Social Science version 22 for Microsoft Windows; SPSS Inc., Chicago, Illinois, USA). The parametric quantitative data were expressed as mean ± SD with the independent t-test was used to compare the means of both groups. Whereas, non-parametric quantitative was expressed as median (range) with the Mann–Whitney U test was used to compare medians of both groups. The Chi-square test was used for comparison of the qualitative data of both groups. A logistic regression analysis was performed to ascertain the effects of dependent variables on the likelihood of developing complications. A *P* value less than < 0.05 was considered significant.

## Results

**Fig. 2:**
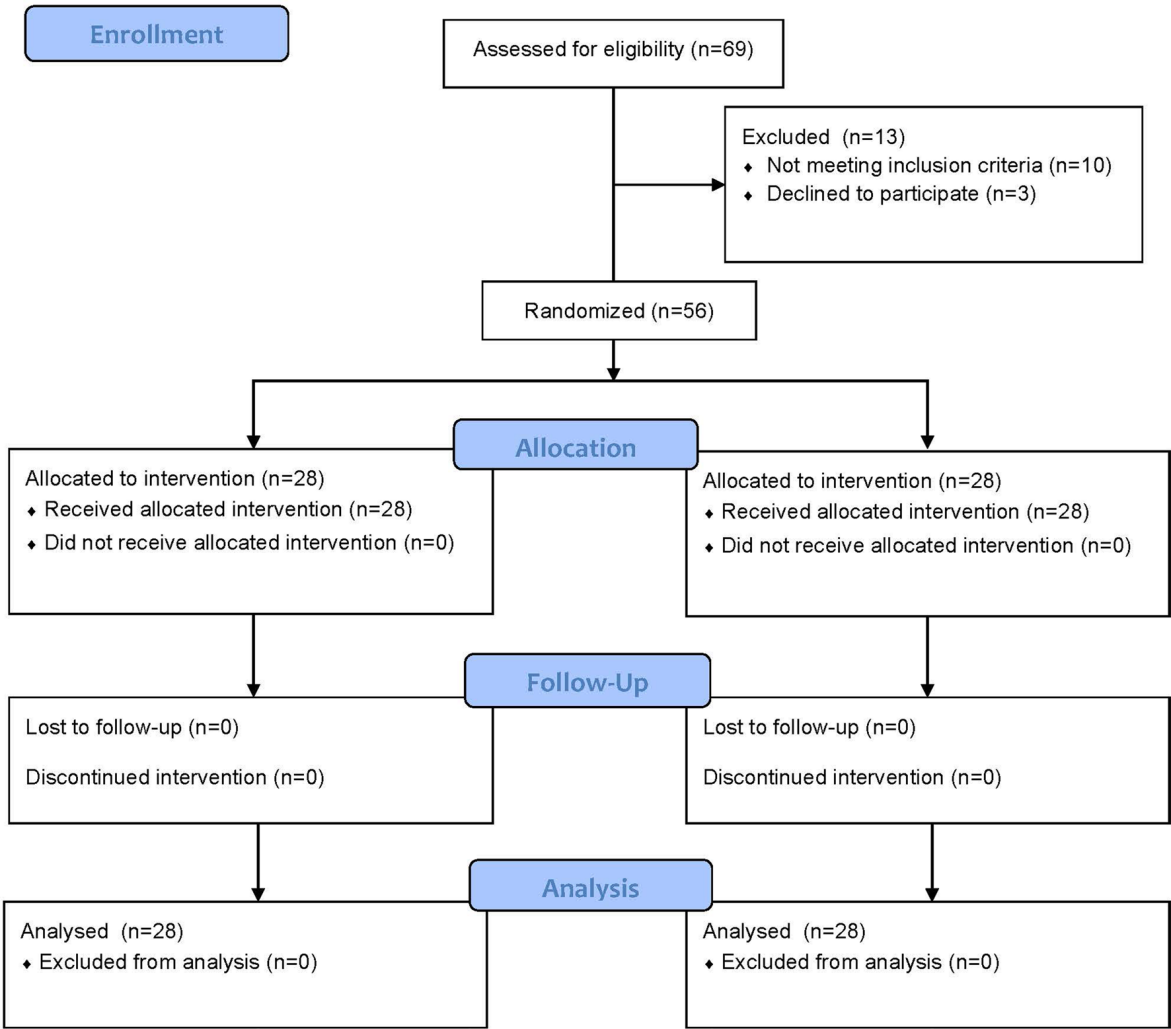
Consort flow chart shows patients’ recruitment process

Fifty-six patients equally divided into two groups were ultimately included in the final analysis. The consort flow chart (Fig. 2) shows the patients' recruitment process. Due to the impact of the COVID-19 pandemic with the suspension of elective clinical visits, follow-up was continued via telephone-call and if recurrence or any other complication suspected, a clinic visit was arranged. A clinic visit was achieved in 16 patients from group A, and 18 patients from group B. The remaining 12 patients from group A and 10 patients from group B were followed up through a telephone-call.

### Patients’ characteristics

Patients were 51 (91.1%) females and 5 (8.9%) males with a mean age of 45.04 ± 8.48 years and a mean BMI of 30.18 ± 3.93 kg/m^2^. Thirty-three (58.9%) patients had ASA score (I), 19 (33.9%) patients had ASA score (II), while 4 (7.1%) patients were of ASA score (III). Twenty-seven (48.2%) patients had accompanying co-morbidities while forty-six (82.1%) patients had prior abdominal surgeries.

Patients were randomly assigned to one of two equal groups; group A underwent placement of mesh only repair technique and group B underwent posterior component separation with transversus abdominis muscle release technique. Table 1 shows the characteristics of patients in both groups. There was no significant statistical difference between the two groups, except for comorbidities.

**Table 1:** Patients’ characteristics

		Total	Group A	Group B	P value
Total		56			
Gender	Female	51 (91.1%)	25 (89.3%)	26 (92.9%)	0.639
	Male	5 (8.9%)	3 (10.7%)	2 (7.1%)	
Age (Mean ± SD) (years)		45.04 ± 8.48	45.32 ± 9.29	44.75 ± 7.74	0.804
BMI (Mean ± SD) (kg/m^2^)		30.18 ± 3.93	30.28 ± 3.83	30.08 ± 4.10	0.854
ASA Score	I	36 (64.29%)	22 (78.6%)	14 (50%)	0.081
	II	16 (28.57%)	5 (17.9%)	11 (39.3%)	
	III	4 (7.14%)	1 (3.6%)	3 (10.7%)	
Co-morbidity	No	29 (51.79%)	19 (67.9%)	10 (35.7%)	0.016
	Yes	27 (48.21%)	9 (32.1%)	18 (64.3%)	
	Hypertension	15 (26.78%)	9 (32.14%)	6 (21.43%)	
	Diabetes	8 (14.28%)	3 (10.71%)	5 (17.86%)	
	BA	5 (8.93%)	0 (0.0%)	5 (17.86%)	
	Liver disease	4 (7.14%)	0 (0.0%)	4 (14.29%)	
	Others	2 (3.57%)	0 (0.0%)	2 (7.14%)	
Prior abdominal surgery	No	10 (17.86%)	7 (25%)	3 (10.7%)	0.163
	Yes	46 (82.14%)	21 (75%)	25 (89.3%)	
	CS	20 (35.71%)	11 (39.29%)	9 (32.14%)	
	Hernioplasty	8 (14.29%)	5 (17.86%)	3 (10.71%)	
	Exploration	7 (12.5%)	5 (17.86%)	2 (7.14%)	
	others	15 (26.79%)	5 (17.86%)	10 (35.71%)	

### Hernia characteristics

The mean duration of symptoms was 14.29 ± 5.5 months. Forty-seven (83.9%) patients complained of primary hernia while 9 (16.1%) patients had a recurrent hernia. The type of hernia was paraumbilical in 25 (44.64%) patients, incisional in 24 (42.86%) patients, others (umbilical and epigastric) in 7 (12.5%) patients***.*** Based on superficial abdominal ultrasonography, the mean diameter of the defect size was 7.99 ± 3.31 cm. There was a single defect in 47 (83.9%) patients, and multiple defects were found in 9 (16.1%) patients.

In group A, the duration of symptoms was 13.50 ± 4.89, and it was 15.07 ± 6.03 in group B. 23 (82.1%) patients complained of primary hernia while 5 (17.9%) patients had a recurrent hernia in group A. In group B, the hernia was primary in 24 (85.7%) patients, and recurrent in 4 (14.3%). The type of hernia was paraumbilical in 14 (50%) patients, incisional in 10 (35.7%) patients, other types (umbilical and epigastric) in 4 (14.2%) patients of group A. 11 (39.3%) patients had a paraumbilical hernia, 14 (50%) patients had an incisional hernia, and other types (umbilical and epigastric) in 3 (10.7%) patients in group B. The two groups had no significant statistical difference regarding the characteristics of hernia as shown in Table 2.

**Table 2:** Hernias’ characteristics

			Group A	Group B	P value
Symptoms duration (Mean ± SD) (months)		14.29 ± 5.5	13.50 ± 4.89	15.07 ± 6.03	0.289
Hernia nature	Primary	47 (83.9%)	23 (82.1%)	24 (85.7%)	0.716
	Recurrent	9 (16.1%)	5 (17.9%)	4 (14.3%)	
Hernia type	Paraumbilical	25 (44.6%)	14 (50%)	11 (39.3%)	0.557
	Incisional	24(42.9%)	10 (35.7%)	14 (50%)	
	Others	7 (12.5%)	4 (14.2%)	3 (10.7%)	
Defect size (Mean ± SD) (cm)		7.99 ± 3.31	7.85 ± 2.58	8.13 ± 3.95	0.756
No. of defects	Single	47 (83.9%)	25 (89.3%)	22 (78.6%)	0.275
	Multiple	9 (16.1%)	3 (10.7%)	6 (21.4%)	

### Operative details

The mean operative time was 131.8 ± 25.2 min in group B versus 120.4 ± 23.2 min in group A. The mean intraoperative blood loss was 135.4 ± 39.5 ml in group B while it was 117.1 ± 27.2 ml in group A.

The size of mesh used was 20 × 25 cm in 16 (57.1%) patients, and 30 × 30 cm in 7 (25%) patients and 15 × 15 cm in 5 (17.9%) patients from group A. In group B, 12 (42.9%) patients had a mesh of size of 30 × 30 cm, 11 (39.3%) patients had a mesh of size of 20 × 25 cm, and 5 (17.9%) patients had a mesh of size of 15 × 15 cm. The median number of drains used was 2 (range 1–3). One drain was used in 4 (14.3%) patients, two drains were used in 24 (85.7%) patients in group A. In 2 (7.1%) patients from group B, one drain was used, while two drains were used in 25 (89.3%) patients, and three drains were used in 1 (3.6%) patient. There was no significant statistical difference between both groups except for blood loss which was found to be less in patients of group A who underwent mesh-only repair (Table 3).

**Table 3:** Operative details

			Group A	Group B	P value
Operative time (minutes)		126.07 ± 24.68	120.4 ± 23.2	131.8 ± 25.2	0.083
Blood loss (ml)		126.25 ± 34.38	117.1 ± 27.2	135.4 ± 39.5	0.05
Size of mesh (cm)	15 × 15 cm	10 (17.86%)	5 (17.9%)	5 (17.9%)	0.326
	20 × 25 cm	27 (48.21%)	16 (57.1%)	11 (39.3)	
	30 × 30 cm	19 (33.93%)	7 (25%)	12 (42.9%)	
Number of drains	Median	2	2	2	0.253
	1	6 (10.71%)	4 (14.3%)	2 (7.1%)	
	2	49 (87.5%)	24 (85.7%)	25 (89.3%)	
	3	1 (1.798%)	0 (0%)	1 (3.6%)	

### Post-operative course; pain score, length of hospital stay, and time for drain removal

The median of the VAS pain score was 7 (range 4–8), while the median of postoperative stay was 2 days (range 1–9). The mean of time till drain removal was 12.52 ± 4.24 days. It was 11.4 ± 4.2 days in patients of group A, while it was 13.7 ± 4.0 in patients in group B. There was no significant statistical difference between the two groups regarding the postoperative course except that the drains were removed earlier in patients of group A (Table 4).

**Table 4:** Post-operative course; pain score, length of hospital stay, and time for drain removal

			Group A	Group B	P value
Pain score (VAS)	Median	7	7	7	0.875
	4	2 (3.57%)	2 (7.1%)	0 (0%)	
	5	5 (8.93%)	3 (10.7%)	2 (7.1%)	
	6	16 (28.57%)	6 (21.4%)	10 (35.7%)	
	7	25 (44.64%)	13 (46.4%)	12 (42.9%)	
	8	8 (14.29%)	4 (14.3%)	4 (14.3%)	
Postoperative stay (days)	Mean	2.8 ± 1.87	2.3 ± 1.5	2.6 ± 2.2	0.945
	Median	2	2	2	
Drain period (days)		12.52 ± 4.24	11.4 ± 4.2	13.7 ± 4.0	0.039

### Postoperative morbidities

The surgical wound was of grade I in 19 (67.9%) patients in group A and 16 (57.1%) patients in group B. Grade II wound was found in six (21.4%) patients from group A and in 5 (17.9%) patients from group B. Three (10.7%) patients in group A had a wound of grade III, and in 7 (25%) patients from group B.

Only one (3.6%) patient from group B had a small hematoma that required bedside drainage while None of the patients from group A developed hematoma postoperatively. In group B, 13 (46.4%) patients developed seroma at the surgical site; all were managed in the outpatient clinic and did not require visiting operative theatre again. Five (17.9%) patients from group A developed seroma which was drained in the outpatient clinic during follow-ups with puncture. In group B, surgical site infection occurred in 7 (25%) patients and was treated with antibiotics after culture and sensitivity tests. Three (10.7%) patients from group A developed surgical site infection that required culture and sensitivity and a prolonged course of systemic antibiotics (Table 5).

**Table 5:** Postoperative morbidities

		Total	Group A	Group B	P value
Wound grade	I	35 (62.5%)	19 (67.9%)	16 (57.1%)	0.378
	II	11 (19.64%)	6 (21.4%)	5 (17.9%)	
	III	10 (17.86%)	3 (10.7%)	7 (25%)	
Hematoma	Yes	1 (1.79%)	0 (0.0%)	1 (3.6%)	0.313
	No	55 (98.21%)	28 (100%)	27 (96.4%)	
Seroma	Yes	18 (32.14%)	5 (17.9%)	13 (464%)	0.022
	No	38 (67.86%)	23 (82.1%)	15 (53.6%)	
Infection	Yes	10 (17.86%)	3 (10.7%)	7 (25%)	0.163
	No	46 (82.14%)	25 (89.3%)	21 (75%)	

According to Clavien–Dindo classification, 23 (82.1%) patients from group A and 21 (75%) patients from group B were of grade I, which means no pharmaceutical or surgical treatment for any deviation from the normal postoperative course was needed. 5 (17.9%) patients from group B only had Grade II, i.e. prolonged pharmaceutical therapy by systemic antibiotics was required. 5 (17.9%) patients from group A and 8 (28.6%) patients from group B were classified as grade IIIa. In these patients, surgical intervention without general anaesthesia to evacuate the developing seroma was warranted. Table 6 shows that there were significant statistical differences between both groups regarding the resulting seroma and Clavien–Dindo classification in the favour of mesh only repair (group A).

**Table 6:** Postoperative complications and Clavien–Dindo classification

	Total	Group A	Group B	P value
I	38 (67.86%)	23 (82.1%)	15 (53.6%)	0.025
II	5 (8.93%)	0 (0.0%)	5 (17.9%)	
III A	13 (23.21%)	5 (17.9%)	8 (28.6%)	

### Follow-up and recurrence

The median of the follow-up period was 17 months (range 11–22) in group A and 13 months (range 11–21) in group B. Recurrence of hernia after surgical repair occurred in 4 (14.3%) patients in group B, and in 1 (3.6%) patient of group A. Table 6 shows that there was a significant statistical difference between the groups regarding the fellow up period. The characteristics of the patients with recurrence of hernia in follow-up are listed in Tables 7 and 8.

**Table 7:** Follow-up and recurrence

		Total	Group A	Group B	Significance
Follow-up in months	Median		17	13	0.004
	Mean	15.48 ± 3.28	16.68 ± 3.14	14.29 ± 3.0	
Recurrence		5	1 (3.6%)	4 (14.3%)	0.160

**Table 8:** Patients with recurrence

	Patient No. 13	Patient No. 27	Patient No. 43	Patient No. 44	Patient No. 49
Sex	F	M	F	F	F
Age (years)	46	39	45	47	44
BMI (kg/m^2^)	33.9	25.8	31.6	36.1	33.9
ASA score	I	III	II	II	II
Comorbidity (s)	No	Liver cirrhosis and myeloid hyperplasia	DM	HTN	Postop. hypothyroidism
Previous abdominal surgery (s)	Mesh repair	Appendectomy	Hysterectomy	Free	No abdominal surgery
Symptoms duration (months)	12	28	19	7	15
Hernia type	Paraumbilical	Incisional	Incisional	Paraumbilical	Incisional
Hernia nature	Recurrent	Primary	Primary	Primary	Primary
Defect size	10 × 5 cm	12 cm	6 × 3 cm	8 cm	2.5 and 4.5 cm
No. of defects	Single	Single	Single	Single	Multiple
Surgery type	MO*	TAR**	TAR	TAR	TAR
Hospital stay (days)	2	1	2	9	1
Mesh size (cm)	20 × 25 cm	20 × 25 cm	15 × 15 cm	30 × 30 cm	20 × 25 cm
Mesh fixation	Sutures	Sutures	Sutures	Sutures	
Operative time (minutes)	160 min	130 min	180 min	180 min	90 min
Blood loss (ml)	150 ml	200 ml	210 ml	200 ml	100 ml
No. of drains	2	1	2	2	2
Drain time (days)	19	12	7	22	9
Skin closure	Sutures	Sutures	Sutures	Sutures	Sutures
Pain score	8	6	7	8	7
Hematoma	No	No	No	No	No
Seroma	Yes	No	No	Yes	No
Infection	Yes	No	No	Yes	No
Wound grade	III	I	I	III	I
CD class***	IIIa	I	I	IIIa	I
Fellow-up (months)	19	14	12	12	12

*Mesh only repair

**Transversus abdominis muscle release technique

***CD classification; Clavien–Dindo Classification

## Discussion

To the best of our knowledge, this the first RCT comparing the PCS with TAR technique to the mesh-only repair for complex VHs. Complex hernias were defined according to Slater et al. [1], 56 patients with complex VHs were included in this study. The causes of the complexity of hernia in our patients were the recurrence of hernia in 9 patients, the presence of multiple hernial defects in 9 patients, and the large size of the defect in 38 patients. There was no significant difference between both groups regarding patient or hernia characteristics except for associated comorbidities.

For the mesh-only repair, we employed sublay-mesh placement which demonstrates improved outcomes compared to onlay, inlay, and underlay repairs. An expert consensus, considered sublay is the optimal mesh location in open elective VHs repair [15]. In a recent meta-analysis, sublay was associated with a lower risk of recurrence and SSI compared to onlay, inlay, and underlay. The pooled recurrence rate was 7.0%, 14.7%, 16.5%, 30.2% for sublay, underlay, onlay, and inlay respectively. The pooled SSI rate was 3.7%, 16.7%, 16.9%, and 31.3% for sublay, underlay, onlay, and inlay respectively [16].

The TAR technique was performed as described by Novitsky et al. [10]. They reported a recurrence rate of 4.7% and wound-related complications rate of 24% when TAR employed in 42 patients with complex VHs. Recently, Hodgkinson et al. [17] in a meta-analysis showed a pooled recurrence rate of 5.7% for 281 patients who received TAR.

In this study the TAR group was associated with increased operative time and estimated blood losses; however, these results were not statistically significant. These results could be attributed to the significant comorbidities associated with this group of patients, and the further dissection needed to perform the TAR technique.

It is not surprising that any innovative surgical technique represents a challenge to surgeons. However, the TAR technique is easy to be implemented for surgeons who operate the Rives-Stoppa technique with the learning curve for TAR should be about 5 cases [18].

Ultimately, the goal of component separation is anterior fascial advancement and restoration of the linea alba. Majumder et al. [19] in their cadaveric model comparing ACS and PCS techniques, reported a significant difference favouring the PCS with 1.4 cm additional fascial advancement anteriorly especially in the upper and mid-abdomen and 2.5 cm additional fascial advancement posteriorly across the upper, mid, and lower abdomen. In a later study, they reported an average of 5.0 ± 0.9 cm baseline fascial advancement achieved only by “Midline Laparotomy”, which considered a reference point after which advancement is measured. Additionally, the “Retrorectus dissection”, the “Transversus Abdominal Division”, and the “Retromuscular Dissection” provided a total gain of 2.6-cm (52%), 3.9 cm (78%), 5.2 cm (104%) increase from baseline respectively [20].

Similar results favouring PCS over ACS were reported by Moores et al. [21], interestingly, they considered the release of posterior lamella of the internal oblique muscles “PLR” as an independent intervention rather than an intermediate step in the TAR procedure, as it achieved 92% of the facial advancement achieved by the TAR.

Safety represents a cornerstone in comparison of different surgical techniques. Overall, only seroma formation was statistically significant favouring the mesh-only group. This could be explained by extensive dissection in the TAR technique. We used the Clavien–Dindo [22] classification as a precise method to report postoperative complications, as it was based on the therapeutic consequences [12]. We found a significant statistical difference in the Clavien–Dindo classification between both groups in the favour of mesh-only repair. However, the results obtained regarding SSI are still similar to those published in TAR literature [14, 23, 24].

Recurrence is a crucial outcome while comparing different surgical technique for hernia repair it could represent the efficacy of the surgical technique. Five patients experienced a recurrence of their hernias, and it was confirmed clinically and radiologically, of these four patients were in the TAR group and one patient in mesh-only group. However, these numbers did not reach a statistically significant difference. During the period of follow-up, none of these patients received additional intervention due to the pandemic with the cancellation of all elective procedures.

The follow-up visit consisted of a thorough physical examination and the pelvi-abdominal US or CT scan if required. Thirty-four (60.7%) patients completed their follow-up schedule at 12-months physically by direct clinic visit. In the other side, the remaining 22 (39.2%) patients completed their follow-up schedule follow-up schedule at 12-months by telephonecall due to far distance from the hospital in 3 patients and due to the pandemic in the remaining 19 patients. None of these patients evaluated through a telephone interview except two reported pain or a possible bulge that required imaging. After clinical and radiological evaluation for those two patients, none was found to have hernia recurrence. Although the telephone interview is not the most accurate method to detect recurrence, it was more convenient and safer during the pandemic, and was adopted by other hernia centres for follow-up based on international recommendations [25–27].

Despite our efforts, this study still limited by certain factors. First, this a single-center experience. Second, the short duration of follow-up, which should be extended. Future multicenter international prospective study recruiting more patients with a longer period of follow-up is needed.

## Conclusions

To conclude, although TAR may be associated with longer operative times and more blood losses, these were not found to be statistically significant. Postoperative complication, except for seroma, and recurrence rates were comparable in both groups. However, a future multicenter prospective study recruiting more patients and of a longer follow-up period is needed to precisely evaluate this promising technique for the treatment of complex ventral hernia.

## Data Availability

The corresponding author will provide any information about the data presented in the article when requested.
